# Histopathological outcome predictors of quiescence/remission in inflammatory bowel diseases: A need being addressed

**DOI:** 10.1002/ueg2.12678

**Published:** 2024-10-24

**Authors:** Vincenzo Villanacci, Gabrio Bassotti

**Affiliations:** ^1^ Institute of Pathology ASST Spedali Civili University of Brescia Brescia Italy; ^2^ Gastroenterology and Hepatology Section Department of Medicine and Surgery University of Perugia Perugia Italy

**Keywords:** claudin‐2, inflammatory bowel diseases, pathology, quiescence, remission

To date, we have numerous therapeutic options to treat patients with inflammatory bowel diseases (IBD), options that have been revolutionized by biological agents and small molecules, drugs that may also be combined to reach better therapeutic goals.^1^

However, the achievement of the ultimate optimal target, histological mucosal healing, is still far from the finish line.^2^ This is likely due to several reasons, but there are few doubts that an accurate pathological evaluation is of paramount importance to achieve this goal, and especially when the treatment results led to clinical and endoscopical quiescence/remission. Therefore, a strong need has arisen to have available better endoscopic and pathologic tools to fill this gap.^3^ Concerning this extremely important topic, the lack of standardization of histology reports and the availability of numerous histological scores, many not validated^4^ or complex to use,^5^ inevitably contribute to “muddy the water.” These considerations are pushing several researchers toward the quest for new, simple diagnostic tools useful to grade the histological activity (especially the minimal, residual or focal one, often missed by non‐dedicated or inexperienced pathologists) not only in the setting of research trials but also for the daily routine purposes.^6^ The recent paper of Zammarchi et al.^7^ tried to fill this gap by showing that epithelial neutrophils and claudin‐2 (an intestinal tight junction protein) expression may predict outcome in IBD patients, most of them in endoscopic remission.

We feel extremely interesting that claudin‐2 expression was able to predict major adverse outcomes, especially in ulcerative colitis patients, since we have recently shown that this expression might be extremely useful to identify both active and focal/residual minimal histological activity in IBD patients.^8^ In fact, claudin‐2 was not expressed in other types of colitides except acute ischemic colitis, and showed excellent interobserver reproducibility and good correlation with histological scores.^8^ Moreover, this expression is easily assessed also by non‐dedicated or less experienced pathologists, and it could help in better assessing IBD biopsies in the daily routine, simplifying the task, especially in high‐volume centers, with a possible overcoming of the use of histological scores that are too complex and subjective.^9^

The study of Zammarchi, in addition to our experience, opens also new pathophysiological issues, confirming the important role of abnormal intestinal permeability among the basic mechanisms of IBD pathogenesis.^10^ In fact, impairment of the intestinal barrier could represent a persisting mechanism of disease activity even in patients in whom complete histological remission has been achieved. If confirmed, this hypothesis could explain the residual activity in a subset of patients, even after appropriate therapeutic measures have been carried out, and address research toward the individuation of different therapeutic approaches able to improve/restore the abnormal intestinal permeability. In fact, we must never forget that we are talking about an organ, the colon, that cannot be put “to rest” and is therefore subjected to significant environmental and dietary influences that can alter the clinical, endoscopic, histological and laboratory picture.

We feel that studies such as those of Zammarchi et al. are important to help paving the road to address in a more targeted manner the lengthy issue of identifying quiescent/residual disease activity in IBD patients. Under such light, the use of claudin‐2 (whose reading adds only a handful of seconds to the daily routine) seems very promising, even though more and larger studies are needed to eventually add this tool to our diagnostic armamentarium and, hopefully, suggest new approaches to the diagnostic/therapeutic approach to IBD, increasing the STRIDE.^11^

## CONFLICT OF INTEREST STATEMENT

The authors have no conflicts of interest to declare.

## Data Availability

The data that support the findings of this study are available on request from the corresponding author. The data are not publicly available due to privacy or ethical restrictions.
